# Supplementary material for: Synchronized stepwise control of firing and learning thresholds in a spiking randomly connected neural network toward hardware implementation
Source: Front Neurosci. 2024 Nov 13;18:1402646. doi: 10.3389/fnins.2024.1402646 (PMC11599226; doi:10.3389/fnins.2024.1402646)
Supplement: Supplementary file 1 [file Data_Sheet_1.PDF]

Supplementary Materials

Synchronized Stepwise Control of Firing and Learning Thresholds in a Spiking Randomly Connected Neural Network toward Hardware Implementation

Kumiko Nomura*†, Yoshifumi Nishi†

† These authors contributed equally to this work and share first authorship

*** Correspondence:**
Kumiko Nomura,
Frontier Research Laboratory, Corporate Research & Development Center, Toshiba Corporation, Komukai-Toshiba-cho 1, Saiwai-ku, Kawasaki 212-8582, Japan

kumiko.nomura@toshiba.co.jp

1 Circuit Configuration of Synapse and SDSP System

Supplementary Figure 1 shows a schematic diagram of crossbar architecture of the synaptic system. Each synaptic unit is located at a crossbar point (Supplementary Figure 1A). Spike signals from a neuron are shared with all synaptic units on the same vertical line. Synaptic current generated at a synaptic unit flows into the connected horizontal line, which is the input line of the corresponding neuron which is depicted at the end of the line on the right. Supplementary Figure 1B shows the circuit diagram of the synaptic unit $U(i,j)$ connecting neuron i and neuron j which consists of three parts: a learning part, a selector part, and a synaptic weight part. When the synaptic unit receives a spike signal, it sends the synaptic weight current (Supplementary Figure 1B) to the next neuron j though line j. When the transmission is completed, SDSP learning is performed in the learning part, and the synaptic weight is controlled by the selector part's signal (Supplementary Figure 1B). The learning part compares the membrane potential V_{mem}^{j} of the neuron j with $V_{SDSP}^{UP}(j)/V_{SDSP}^{DOWN}(j)$ when the neuron i fires, and sends a control signal to the next selector part based on the comparison result, respectively. The selector part decides which weight selects using the results of the learning part. Since $U(i,j)$ outputs four different weight currents, the selector part consists of four multiplexers and four flip-flops. Notice that if $V_{SDSP}^{UP}(j) = V_{SDSP}^{DOWN}(j)$, the learning unit can be integrated with one comparator which is a minimum configuration. Furthermore, when the weight is binary, the learning unit can be integrated with two multiplexers and flip-flops, which is the minimum configuration.

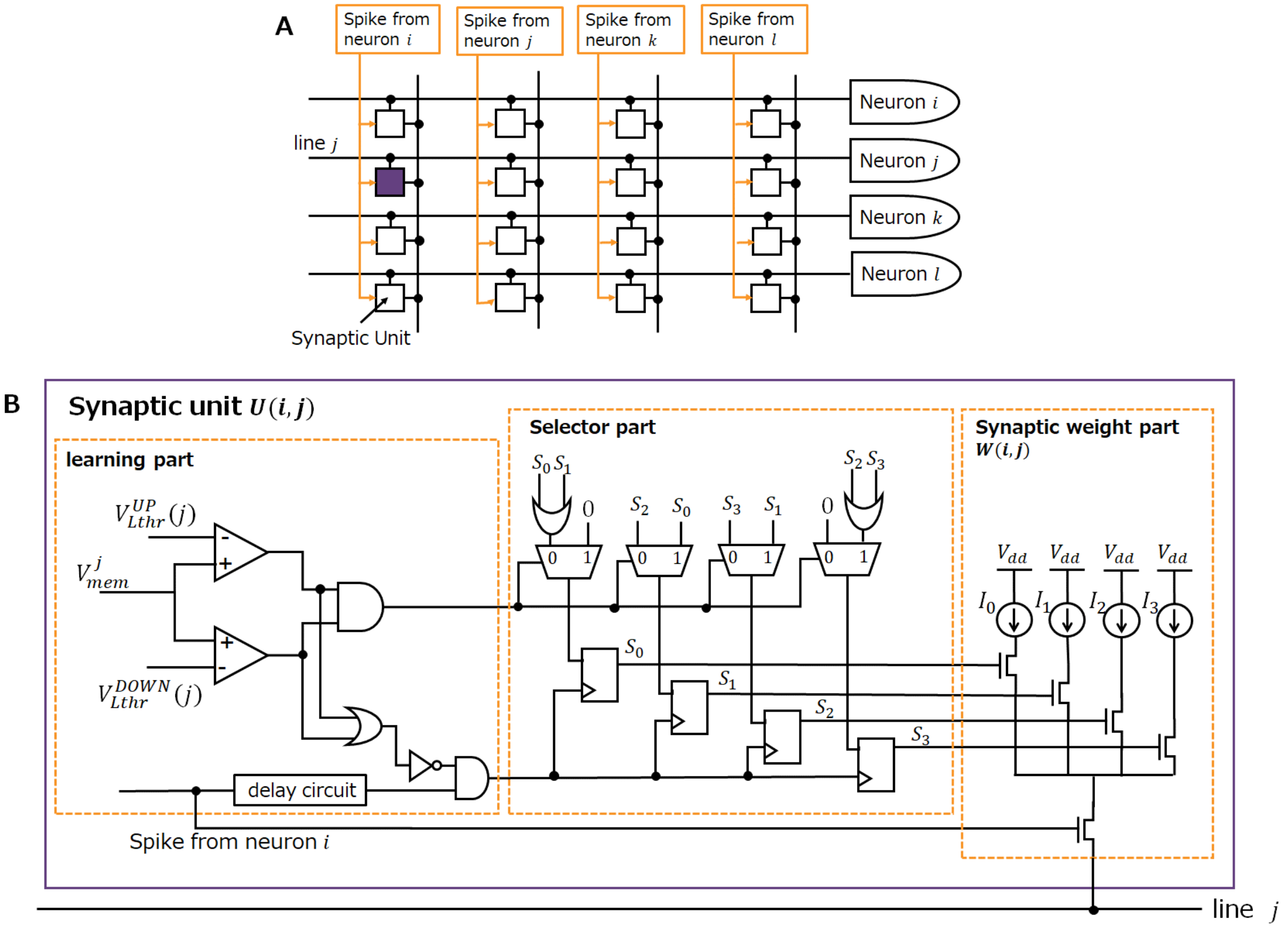

Supplementary Figure 1. Schematic diagram of crossbar architecture using synaptic unit with SDSP. (A) Construction of crossbar architecture. (B) Structure of Synaptic unit.

2 Circuit Configuration of Synaptic Synchronization IP System

Supplementary Figure 2 shows a schematic diagram of a LIF neuron with our proposed IP. This IP system operates by an output spike of the LIF neuron and outputs a firing threshold V_{thr} and SDSP thresholds $V_{Lthr}^{UP/DOWN}(j)$. The IP system consists of a frequency calculation part (Supplementary Figure 3A), a threshold determination part (Supplementary Figure 3B), and a threshold output part (Supplementary Figure 3C).

The total number of selectors and flip-flops in the threshold determination part are determined by a maximum threshold value, a minimum threshold value, an initial threshold value, and LR_{thr}. Therefore, the circuit area of the IP system depends on the threshold determination part.

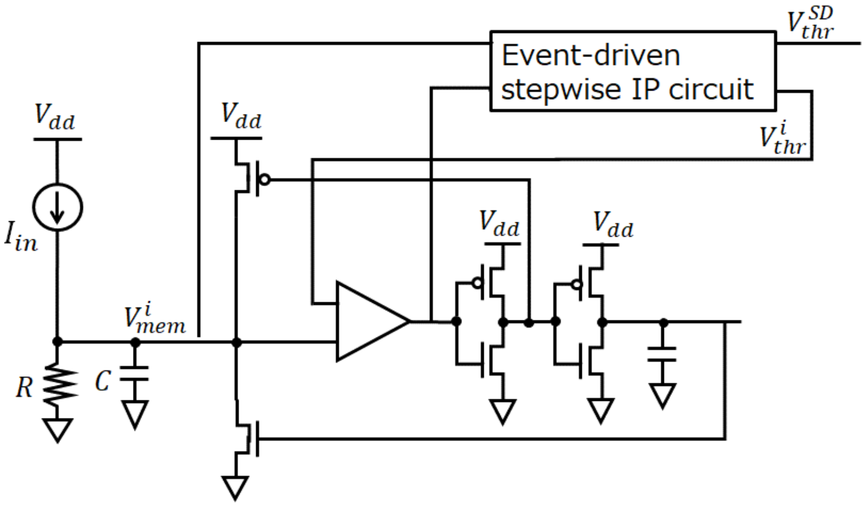

Supplementary Figure 2. Schematic diagram of neuron circuit with event-driven stepwise IP.

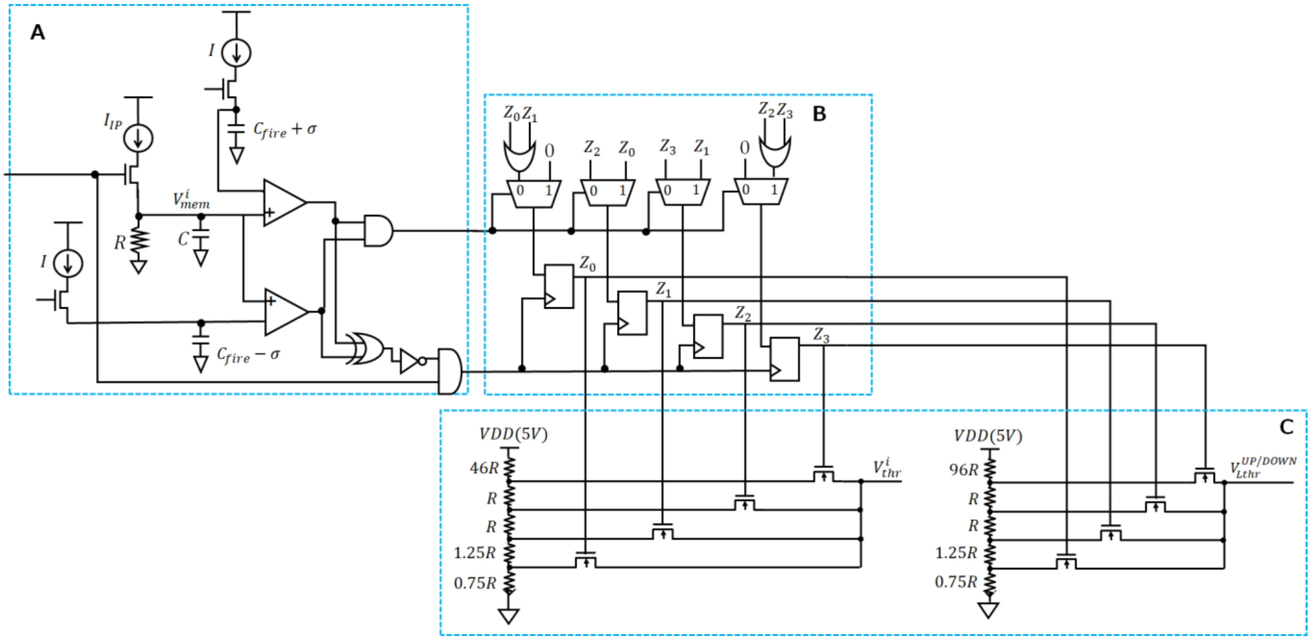

Supplementary Figure 3. Schematic diagram of the event-driven stepwise IP. **(A)** the frequency calculation part. **(B)** the threshold determination part. **(C)** is the threshold output part.

3 Relationship between σ and an anomaly detection capability

Supplementary Figure 4 shows the Δ_{thr} against σ. From this figure, when the when LR_{thr} is large, a M-SRNN with high detection capability can be reconstructed if σ is above a certain value. Conversely, when LR_{thr} is small, the M-SRNN with high detection capability can be reconstructed if σ is below a certain value. Therefore, if LR_{thr} is increased, it is necessary to select large σ to reconfigure the M-SRNN with high detection capability.

A C_{fire} should converge to C_{IP} by IP operation. In other words, it is important to perform the IP operation only when the inequation (S-1) is satisfied.

$$\left|C_{fire}^{new} - C_{IP}\right| < \left|C_{fire} - C_{IP}\right| \qquad \cdots \text{(S-1)}$$

where an indicator of activity C_{fire} before the IP operation and an indicator of activity C_{fire}^{new} after the operation.

When LR_{thr} is large, C_{fire}^{new} changes significantly. Therefore, when $\left|C_{fire} - C_{IP}\right|$ is small, the condition in (S-1) will not be satisfied. Therefore, as in Supplementary Figure 4, an M-SRNN with high detection accuracy can be reconstructed by setting the suitable σ depending on the LR_{thr}.

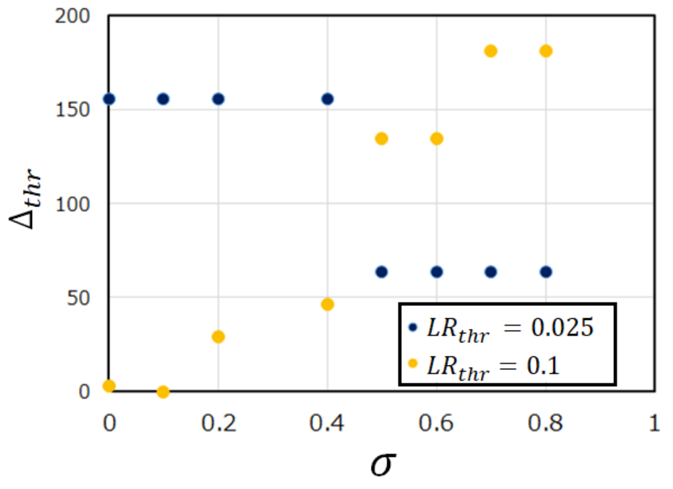

Supplementary Figure 4. Δ_{thr} with $LR_{SDSP} = 2.0$ and $LR_{thr} = 0.025V$ or $0.1V$ against σ.

4 Simulation Results for Counting Task Benchmark

4.1 Counting Task Benchmark

To demonstrate the effectiveness of our method, we use the same benchmark task as those in the previous papers (Lazar et al., 2009; Payvand et al., 2022) called a counting task, in which a network learns two types of sequence of characters $S_1^n = [A, B, \cdots, B, C]$ and $S_2^n = [D, E, \cdots, E, F]$, where the characters B and E are repeated n times consecutively. The goal of the task is to predict the next input character from a partly inputted sequence (Supplementary Figure 5). For precise prediction, the network must be able to count how many Bs and Es are repeated.

Six input neurons are prepared for the 6 characters. When a character is inputted, the corresponding neuron fires at the Poisson frequency of $F_{input} = 100Hz$. The network is asked to distinguish $n = 10$ repetitions of B and E. The position of each character in the two sequences is assigned to one different output neuron, whose activity represents the prediction of the network.

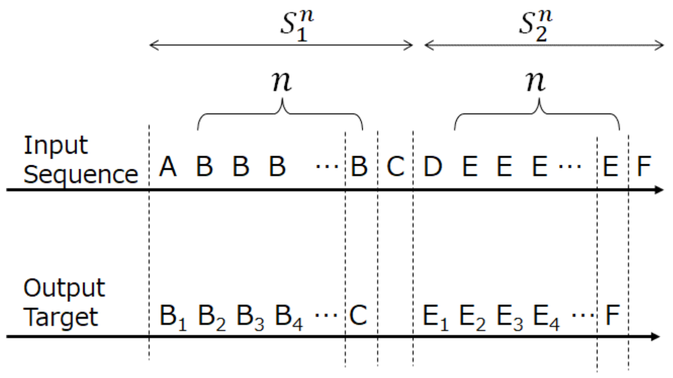

Supplementary Figure 5. Counting Task benchmark in the simulation. Predicted result of the correct answer at the output, corresponding to each input sequence.

4.2 Simulation parameters

The effectiveness of our M-SRNN model with IP and SP explained above is evaluated using Brian simulator (Goodman and Brette, 2008) by Counting Task Benchmark (Lazar et al., 2009; Payvand et al., 2022) with parameters listed in Supplementary Table 2. In the simulation, the learning step LR_{SDSP} and the firing threshold change width LR_{thr} are selected from $S_{LR} = \{0.01, 0.02, 0.05, 0.1, 0.2, 0.5\}$ and$P_{thr} = \{0.025\ V, 0.05\ V, 0.1\ V,\ 0.3\ V\}$, respectively. The ranges of W and V_{thr} are $0 \leq W \leq 2$ and $0.125\ V \leq V_{thr} \leq 0.4\ V$. With regard to the SP synchronization with IP, we set $V_{Lthr}^{UP}(i) = V_{Lthr}^{DOWN}(i) = V_{thr}^{i}/2$ throughout this work, hence LR_{Lthr}^{UP}=$LR_{Lthr}^{DOWN} = LR_{thr}/2$. All initial synaptic weights between excitatory neurons are set to 1.0, and the initial firing threshold is set to 0.2V for all neurons. All other synaptic weights are set randomly.

Neurons			Synapses	
	Excitatory	Inhibitory	W	1.0
# of Neurons	160	40	SRNN	
$R\ (M\Omega)$	400	400	P_{EE}	2%
$C\ (pF)$	10	10	P_{II}	0%
$\tau_{Ca}\ (ms)$	100	100	P_{EI}	2%
$V_{th}\ (V)$	0.2	0.2	P_{IE}	10%
C_{ip}(#of fires /sec)	10	-		
$\tau_{ip}(ms)$	100	100		

Supplementary Table 1. Initial values in SRNN simulations.

4.3 Simulation Results

Effectiveness of the proposed method

The results of the Counting Task benchmark are shown in Supplementary Figures 5 for various IP conditions. Note that SDSP is used for all the cases. Supplementary Figure 5A shows the accuracy evolution with the learning epoch for each learning condition of M-SRNN with 200 neurons and $LR_{SDSP} = 0.05$. At each epoch, one of the two sequences is randomly selected and given to the network. For the initial M-SRNN (epoch 0), the accuracy is only 30%, indicating that the untrained network

cannot count the repeated characters. However, the accuracy becomes higher as the learning proceeds. It is clearly observed that M-SRNNs trained with both IP and SDSP result in much better accuracies than that with SDSP only. Figure 5B shows the accuracy against LR_{SDSP} for each LR_{thr}. From the figure, we observe that the accuracy increases as LR_{SDSP} becomes smaller. However, the accuracy reaches only 80% even when LR_{SDSP} is lowered to 0.01 without IP (i.e. with SDSP only). If we are to achieve the accuracy over 90%, IP should be introduced. Therefore, employing IP in addition to SDSP is effective for reconstructing M-SRNN.

Supplementary Figure 5C shows the accuracy against n using M-SRNN of 200 neurons reconstructed with $LR_{SDSP} = 0.05$. From this figure, the M-SRNN can maintain more than 90% prediction accuracy up to $n = 10$ by reconstruction, but beyond that, it is difficult to maintain prediction accuracy. Therefore, in this learning method, the maximum 10 character can be counts using the reconstructed M-SRNN regardless of LR_{thr}. Supplementary Figure 5D shows the accuracy against the number of neurons in M-SRNN trained with $n = 10$ and $LR_{SDSP} = 0.05$. This figure shows that even with small IP resolution, it is possible to reconstruct an M-SRNN with 90% or better accuracy if the network size is 200 neurons or larger.

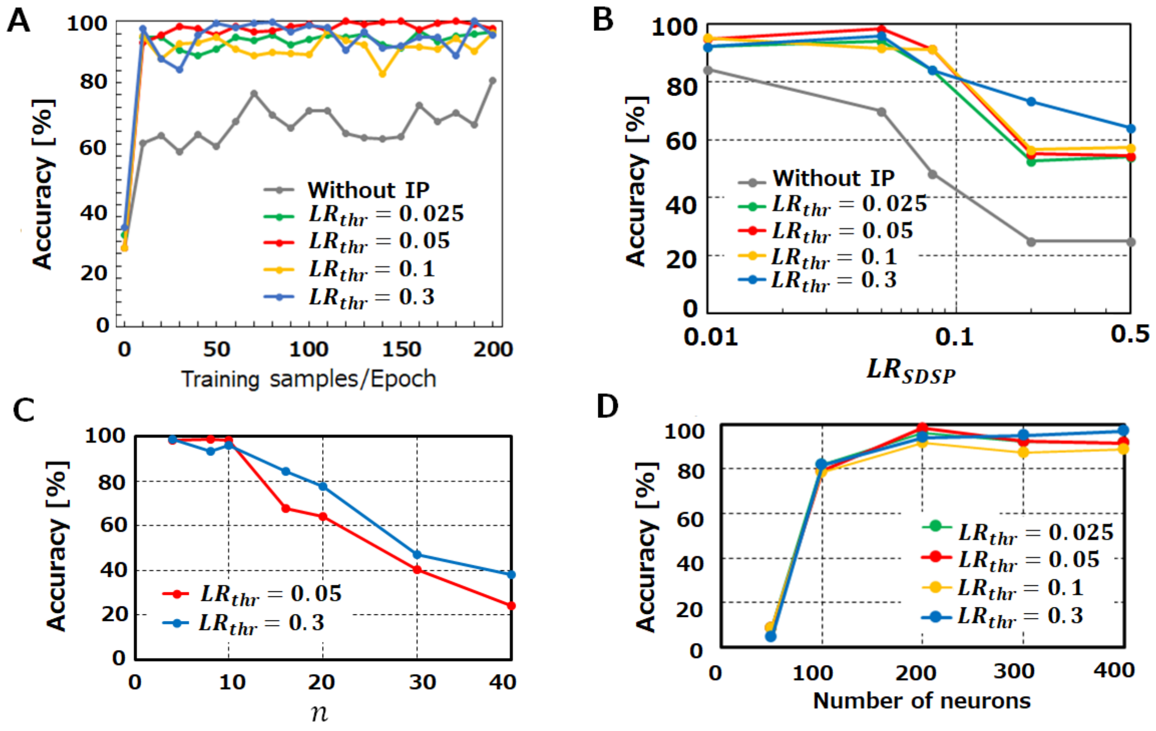

Supplementary Figure 5. The Simulation results in Counting Task benchmark: (A) Accuracy evolutions with the number of training epoch. The accuracy against (B) LR_{STDP}, (C) n, and (D) the number of neurons in M-SRNN.

Relationship between C_{IP} or σ and an elapsed time recognition property

Supplementary Figure 6 shows the accuracy against parameters C_{IP} and σ in case of $n = 10$ and $LR_{SDSP} = 0.05$. Supplementary Figure 6A shows the accuracy against C_{IP} in each LR_{thr}. In order to reconstruct a M-SRNN with highly accuracy, the C_{IP} must be set carefully. From this figure, the

accuracy is the same trend for C_{IP} regardless of LR_{thr}. The difference of the accuracy between the optimal C_{IP} and the others becomes larger if the resolution of LR_{thr} is small. However, there is little difference in accuracy in M-SRNN reconstructed with optimal C_{IP}. Therefore, if C_{IP} is set optimally, it is possible to reconstruct M-SRNN with high accuracy even if the resolution of LR_{thr} is decreased. It is clear that C_{IP} is a very important factor for reconstructing high accuracy M-SRNN. Supplementary Figure 6B shows the accuracy against σ for $LR_{thr} = 0.025$ or 0.3 with $C_{IP} = 10$. As discussed in Supplementary Section 2, the optimal σ and accuracy have also the same trend in the Counting Task benchmark.

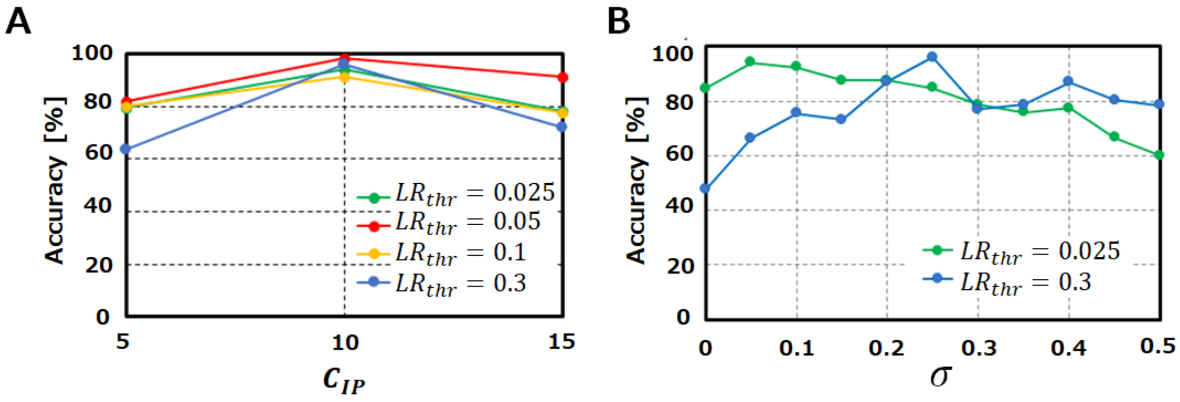

Supplementary Figure 6. The accuracy against learning parameters (A) C_{IP} and (B) σ.

5 Simulation Results for ECG Benchmark

5.1 Effectiveness of IP and SDSP

Supplementary Figure 7 shows anomaly detection results of the original M-SRNN, the M-SRNN reconstructed with SP and that with both SP and IP for ECG waveform No. 14046. Supplementary Figure 7A, C and E show the difference $D(k)$ in the three cases respectively, when a normal ECG waveform in Supplementary Figure 7G is used as a test data. Supplementary Figure 7B, D, and F show the difference $D(k)$ when an abnormal ECG waveform in Supplementary Figure 7H is used. It can be seen in Supplementary Figure 7B that the initial M-SRNN cannot detect anomalous points because D_{thr}^{ab} is almost the same as D_{thr}^{no}, hence no window Δ_{thr}. On the other hand, the M-SRNN reconstructed by SDSP shows clear Δ_{thr}, as shown in Supplementary Figure 7D. Furthermore, if we add the IP function, we obtain larger D_{thr}^{ab} while D_{thr}^{no} is the same, hence larger Δ_{thr}.

The effectiveness of our proposed method is not limited to No. 14064. Supplementary Figure 8 shows anomaly detection results for other ECG benchmarks (No. 14172 and 14134). Supplementary Figure 8A and C show the difference $D(k)$ for the initial and trained M-SRNN respectively, when the abnormal ECG waveform No. 14172 in Supplementary Figure 8E is used as a test data. The same goes for Supplementary Figure 8B, D and F in the case of No.14134. We observe that the initial M-SRNN cannot detect anomalous points because D_{thr}^{ab} is almost the same as D_{thr}^{no} (Supplementary Figure 8A and B), but the trained M-SRNN enlarge Δ_{thr} hence anomaly detection is possible (Supplementary

Figure 8C and D). These results clearly show the effectiveness of the proposed SP and IP models in improving the anomaly detection performance. In particular, if the M-SRNN is reconstructed by using the synchronized SP and IP, a sufficient margin Δ_{thr} is obtained for anomaly detection without misdetection of normal data.

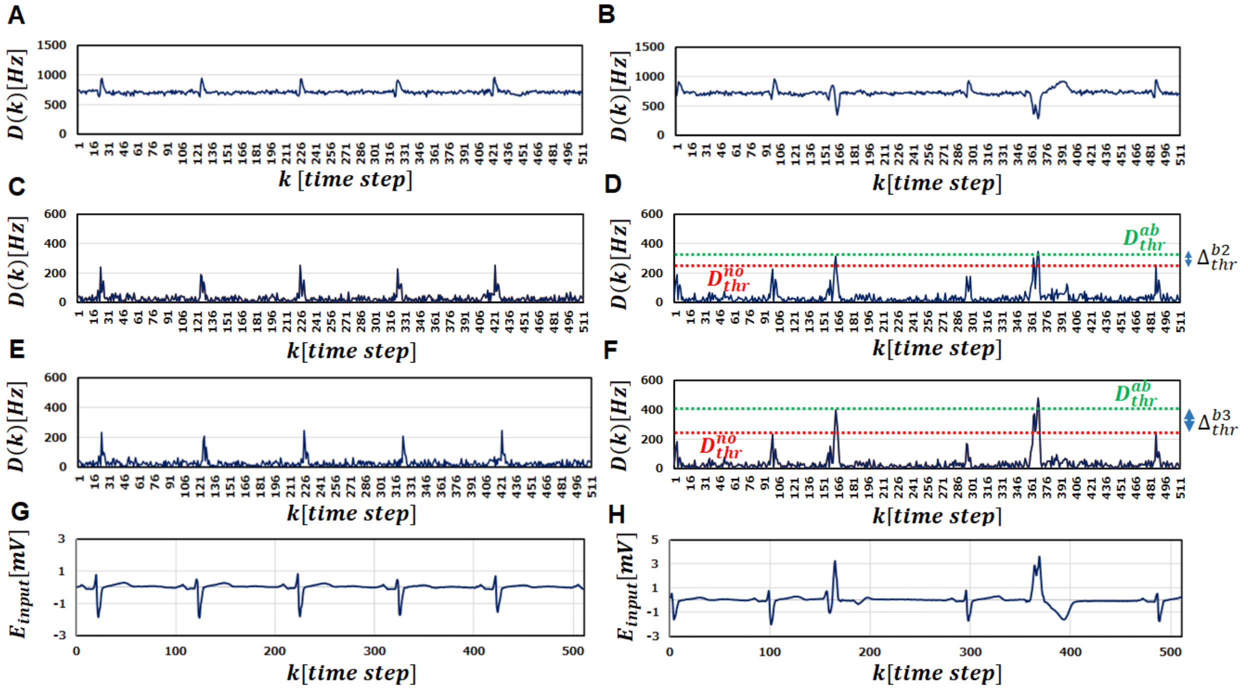

Supplementary Figure 7. $D(k)$ with $T_{bin} = 150\ ms$, $LR_{SDSP} = 2.0$, $LR_{thr} = 0.025\ V$, and $\sigma = 0.3$. **(A)(B)** Normal and abnormal cases by initial M-SRNN, **(C)(D)** by M-SRNN reconstructed by only SDSP learning, **(E)(F)** by M-SRNN reconstructed by SDSP and synchronized IP learning, and **(G)(H)** original data waveform No. 14046, respectively.

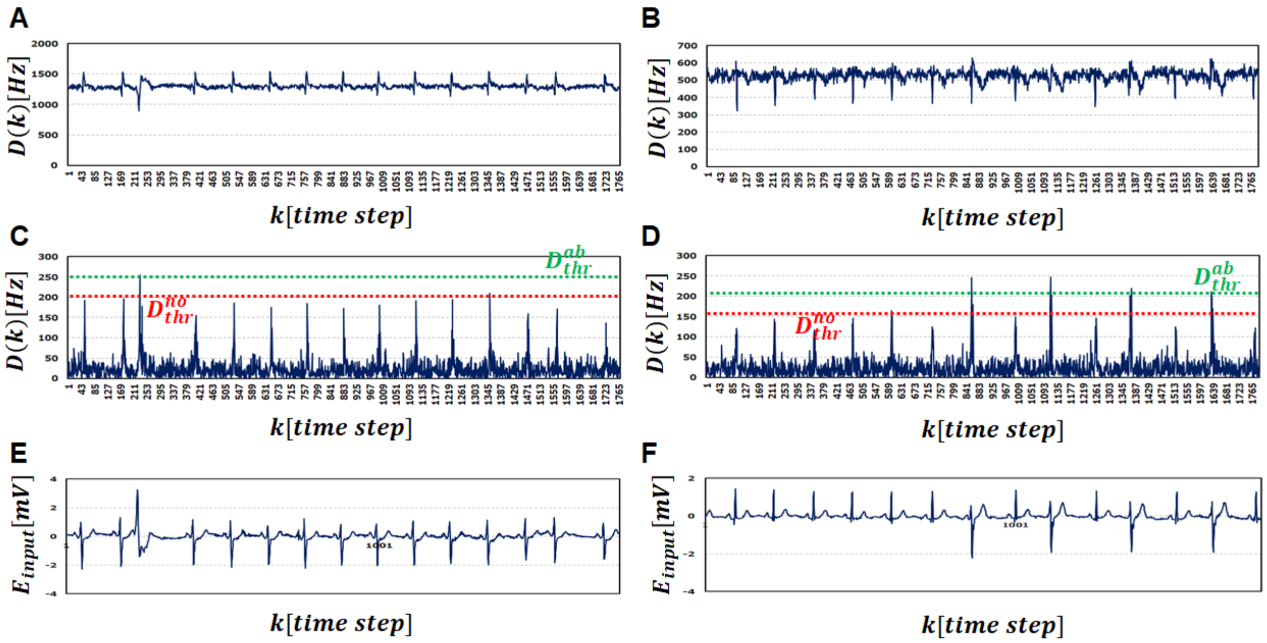

Supplementary Figure 8. $D(k)$ with $T_{bin} = 150\ ms$, $LR_{SDSP} = 1.0$, $LR_{thr} = 0.025\ V$, and $\sigma = 0.3$. **(A)(B)** Abnormal cases (No. 14172 and 14134) by initial M-SRNN, **(C)(D)** by M-SRNN reconstructed by SDSP and synchronized IP learning, **(E)(F)** original data waveforms (No. 14172 and 14134, respectively).
